# Serum Periostin May Help to Identify Patients with Poor Collaterals in the Hyperacute Phase of Ischemic Stroke

**DOI:** 10.3390/diagnostics12081942

**Published:** 2022-08-11

**Authors:** Dora Spantler, Peter Csecsei, Katalin Borocz, Timea Berki, Laszlo Zavori, Attila Schwarcz, Gabor Lenzser, Tihamer Molnar

**Affiliations:** 1 Department of Anaesthesiology and Intensive Care and Department of Neurosurgery, Medical School, University of Pecs, 7624 Pecs, Hungary; 2 Department of Neurosurgery, Medical School, University of Pecs, 7624 Pecs, Hungary; 3 Department of Immunology and Biotechnology, Medical School, University of Pecs, 7624 Pecs, Hungary; 4 Salisbury NHS Foundation Trust, Salisbury SP2 8BJ, UK

**Keywords:** hyperacute ischemic stroke, periostin, ASPECT, collaterals

## Abstract

Background: Periostin is a glycoprotein that mediates cell functions in the extracellular matrix and appears to be a promising biomarker in neurological damage, such as ischemic stroke (IS). We aimed to measure serum periostin levels in the hyperacute phase of ischemic stroke to explore its predictive power in identification of patients with poor collaterals (ASPECT < 6). Methods: We prospectively enrolled 122 patients with acute ischemic stroke within the first 6 h after onset. The early ischemic changes were evaluated by calculating ASPECT score on admission using a native CT scan. An unfavorable outcome was defined as the modified Rankin Scale (mRS) > 2 at 90 days follow-up. Blood samples were collected on admission immediately after CT scan and periostin serum concentrations were determined by ELISA. Results: The admission concentration of serum periostin was significantly higher in patients with unfavorable outcome than in patients with favorable outcome (615 ng/L, IQR: 443–1070 vs. 390 ng/L, 260–563, *p* < 0.001). In a binary logistic regression model, serum periostin level was a significant predictor for ASPECT < 6 status on admission, within 6 h after stroke onset (OR, 5.911; CI, 0.990–0.999; *p* = 0.015). Conclusion: Admission periostin levels can help to identify patients who are not suitable for neurointervention, especially if advanced neuroimaging is not available.

## 1. Introduction

Ischemic stroke (IS) accounts for approximately 80% of all stroke cases and causes a tremendous burden on health resources and families [1]. Inflammation plays a pivotal role in the pathogenesis of IS exerting both beneficial and detrimental effects. The activation of resident cells, such as microglia, astrocytes, and endothelial cells, is neuroprotective and promotes brain regeneration and recovery, whilst the recruitment of immune cells expressing inflammatory mediators and leading to blood–brain barrier (BBB) disruption results in neuronal death, brain edema, and hemorrhagic transformation [2].

Periostin is a 93-kDa secreted N-glycoprotein that mediates cell-matrix interactions and cell functions in the extracellular matrix [3] and has been identified in many pathological conditions such as cardiovascular diseases, tumors, and airway inflammation [4,5,6]. Generally, periostin is expressed at low level in human tissues but can be rapidly upregulated by various pathophysiological signals [7]. Previous studies have revealed that periostin is rapidly secreted into ischemic tissue after acute myocardial infarction and has an essential role in the repair process [5,8]. Additionally, the neuroprotective and neurogenic effects of exogenous periostin have been demonstrated on both in vitro and in vivo IS models [9]. In patients with aneurysmal subarachnoid hemorrhage, a higher admission serum level of periostin was correlated with unfavorable 90-day outcome, worse admission neurological status, larger hemorrhage volume and more frequent development of delayed cerebral ischemia [10]. He et al. have shown that increased serum periostin levels after IS might have been associated with higher National Institute of Health Stroke Scale (NIHSS) scores and more extensive stroke volume in patients with large-artery atherosclerotic stroke [11]. Several studies have proven that the ASPECT score is a simple and effective tool for assessing the collateral system by helping to estimate the core-penumbra ratio and thus the extent of the collateral network [12,13,14,15]. Based on the analysis of a large stroke register database, we know that better collaterals were associated with lower core volumes and higher ASPECT score, but not with higher penumbra volumes [16]. This suggests a major role of collaterals in early tissue loss. All the clinical studies that serve as the basis for the treatment of acute ischemic stroke lasting longer than 6 h [16,17] used core-based selection when conducting the study. Until now, there hasnot been a single clinically applicable serum biomarker that would indicate the core volume with high specificity and sensitivity and thus playing a role in clinical decision-making.

In the present study, we aimed to measure serum periostin levels at admission in patients with acute ischemic stroke and investigate whether it has predictive value or decision-making power in very early ischemic stroke by helping to estimate core volume.

## 2. Materials and Methods

### 2.1. Participants

**Figure 1:**
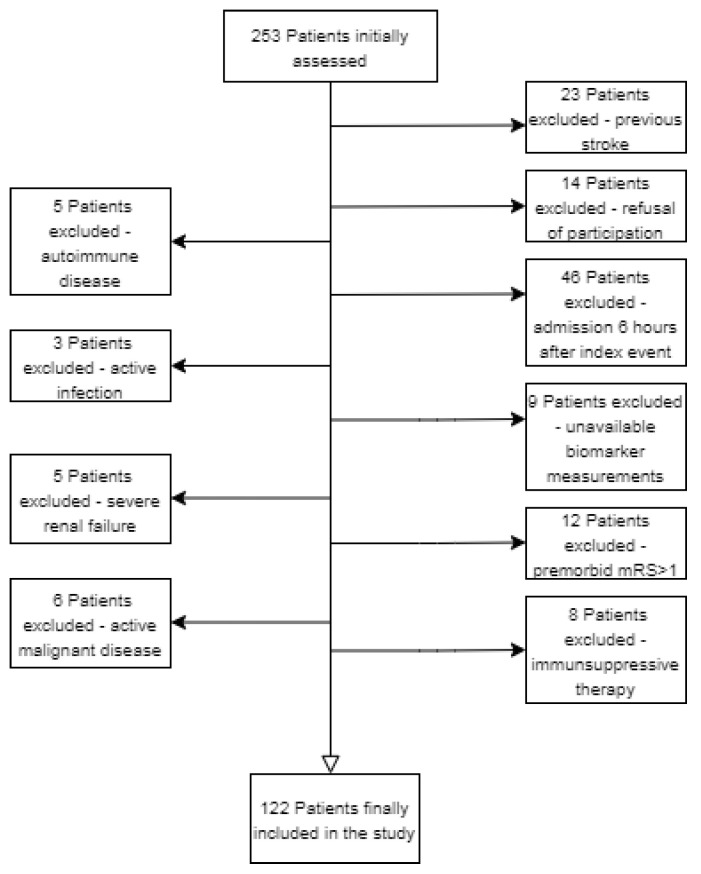
Flow chart illustrating excluded and included patients with acute ischemic stroke.

This was a prospective observational study from a tertiary stroke treatment center in Pecs, Hungary. During the period between July 2019 and April 2021, a total of 122 patients with acute ischemic stroke within the first 6 h after onset were prospectively enrolled. Acute ischemic stroke was diagnosed according to WHO criteria [9]. As controls, we recruited fifteen age- and sex-matched healthy individuals. The following inclusion criteria were applied: (i) first-ever ischemic stroke, (ii) admission within 6 h after the index event. Exclusion criteria were as follows: (i) <18 years; (ii) previous ischemic or hemorrhagic stroke; (iii) premorbid modified Rankin score (mRS) > 1; (iv) active malignant or autoimmune disease; (iv) immunosuppressive therapy; (v) acute or chronic infection at study enrollment; (vi) severe hepatic or renal insufficiency; (vii) and pregnancy. A flow chart of the study is shown in Figure 1. All procedures were performed in accordance with ethical guidelines of the 1975 Declaration of Helsinki. The study was approved by the Hungarian Medical Research Council. Written informed consent was given by each patient or their representatives before enrollment into the study.

### 2.2. Clinical Protocol

Stroke severity was assessed using Glasgow Coma Scale (GCS) and NIHSS scores. The early ischemic changes were evaluated by ASPECT score calculated on admission using native cranial CT (NCCT) scan by a certified neuroradiologist who was blinded to the patients’ clinical information. Unfavorable outcome was defined as an mRS score > 2 at 90 days after IS. Venous blood samples were collected on admission to stroke unit immediately after NCCT scan, but not later than 6 h after symptom onset. The patients received the standard stroke care: (i) within 4.5 h, if there were no contraindications, they received intravenous systemic recombinant tissue plasminogen activator (rtPA); if the possibility of large vessel occlusion arose (NIHSS > 8), CT angiography was performed; if a large vessel occlusion was confirmed (middle cerebral artery—M1, internal carotid artery or basilar artery), thrombectomy was also performed with (ii—EVT + rtPA) or without (iii—EVT alone) systemic thrombolysis. Patients with an ASPECT score < 6 on admission were considered to have a poor collateral network [13].

### 2.3. Laboratory Analysis

The blood samples were immediately centrifuged at 3500 r/min for 15 min and aliquots of other samples were immediately stored at −80 °C before assay. Biomarker concentrations were measured by investigators blinded to the clinical outcome and neuroimaging findings. The serum periostin level was determined by the workers of the Department of Immunology and Biotechnology, University of Pecs, using ELISA-based kits manufactured by Shanghai YL Biotech Co., Shanghai, China. Samples were all processed by the same laboratory technician using the same equipment and blinded to all clinical data. The detection limit for the assay was 0.251 ng/L.

### 2.4. Statistical Analysis

Data were evaluated using SPSS (version 11.5; IBM, Armonk, NY, USA). Categorical data were summarized by means of absolute and relative frequencies (counts and percentages). The Kolmogorov–Smirnov test was applied to check for normality. The chi-square test for categorical data and Student’s *t*-test as well as Mann–Whitney test for continuous data were used for the analysis of demographic and clinical factors. Non-normally distributed data are presented as median and interquartile range. Correlation analysis was performed calculating Spearman’s correlation coefficient(r). To find an independent predictor of ASPECT < 6, a binary logistic regression was used. Receiver operating characteristic (ROC) curve analysis was performed to evaluate the predictive values of serum periostin concentrations for 90-day unfavorable outcome. Subsequently, area under the curve (AUC) and the corresponding 95% CI were calculated. In a combined logistic-regression model, we estimated the additive benefit of periostin concentrations to NIHSS scores. A *p*-value < 0.05 was considered statistically significant.

## 3. Results

### 3.1. Clinical Characteristics

**Figure 2:**
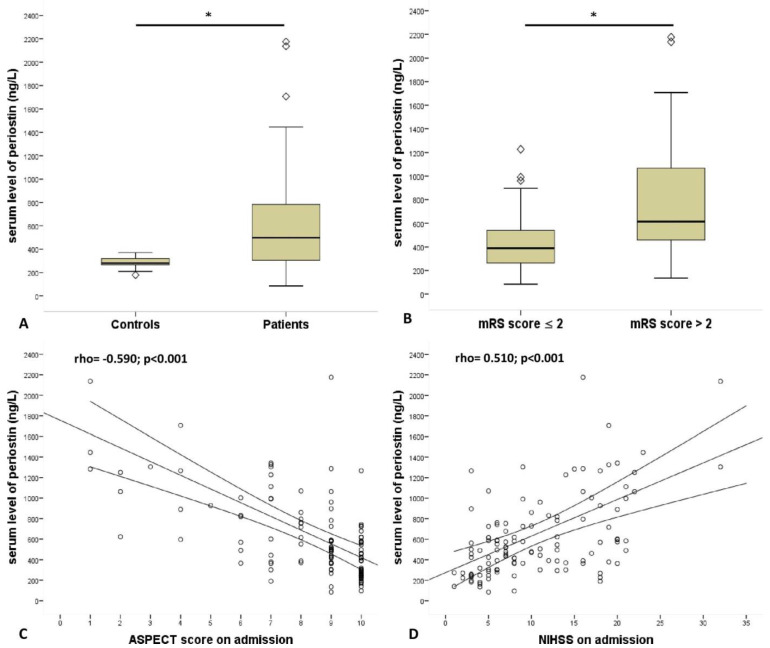
Comparison of serum periostin level (**A**) among patients with stroke and controls, (**B**) between patients with favorable outcome (mRS ≤ 2) vs. unfavorable outcome (mRS > 2) on Day 90 follow-up. Correlation of admission serum periostin level with (**C**) ASPECT score measured on admission and (**D**) NIHSS score recorded on admission. Serum periostin level was measured at 24 h after stroke onset. * *p*-value < 0.001. ASPECT, Alberta stroke program early CT score; mRS, modified Rankin score; NIHSS, National Institutes of Health Stroke Scale. Statistical analysis were performed with Mann–Whitney U-test (**A**,**B**) and using Spearman rank correlation (**C**,**D**).

This cross-sectional study enrolled 122 patients with first-ever acute ischemic stroke. A flow chart of the study is shown in Figure 1, and Table 1 shows clinical profiles of patient groups based on their best mRS scores at 3-month as primary outcome measure. The median age of the patients was 71 years (interquartile range: 63–79, min-max. values: 30–91) and 39.3% were female. Fifteen healthy volunteers served as age-matched normal controls. The median age of controls was 66 (interquartile range (IQR): 55–73, range 46–82), and 46.7% were female. The age and sex differences between patients and controls were non-significant. Regarding comorbidities, 100 patients (82%) had hypertension, 35 patients (29%) had diabetes mellitus, and 34 patients (27.9%) presented with atrial fibrillation (AF). The median admission NIHSS score was 8 (IQR: 5–16, min-max: 1–32), and the median systolic and diastolic blood pressure on admission were 150 mmHg (IQR: 130–170, range: 90–240) and 84 mmHg (IQR: 80–93.5, range: 48–118). The median ASPECT score was 9 (IQR: 7–10, min-max: 1–10). In total, 29 patients (24%) underwent endovascular mechanical thrombectomy (EVT), 51 (42%) received intravenous systemic recombinant tissue plasminogen activator (rtPA) treatment, and 17 (14%) underwent a combined EVT + rtPA treatment. A total of 25 patients (20.5%) were not eligible neither for EVT nor for rtPA; therefore, they received conservative treatment. The median serum level of periostin was 498.4 ng/L (IQR, 305–783) in patients with IS, and 280.4 ng/L (IQR, 259–332) in healthy controls (*p* < 0.001, Figure 2A).

### 3.2. Admission Periostin Level, Comorbidities and Outcome

The admission serum periostin concentration was significantly higher in those patients who later had an unfavorable 90-day outcome compared to patients with favorable outcome (Figure 2B and Table 1). Moreover, the admission concentration of serum periostin showed an inverse correlation with ASPECT score (Figure 2C), while it was positively correlated with admission NIHSS score (Figure 2D). In multivariate analysis, we found positive associations between admission level of periostin and atrial fibrillation, admission white blood cell (WBC) count, neutrophile–lymphocyte ratio (NLR), creatinine, C-reactive protein (CRP), and glucose level (Table 2). In contrast, serum periostin level was negatively associated with GCS and ASPECT score, both measured at admission.

### 3.3. Variables Associated with Poor Collaterals

**Figure 3:**
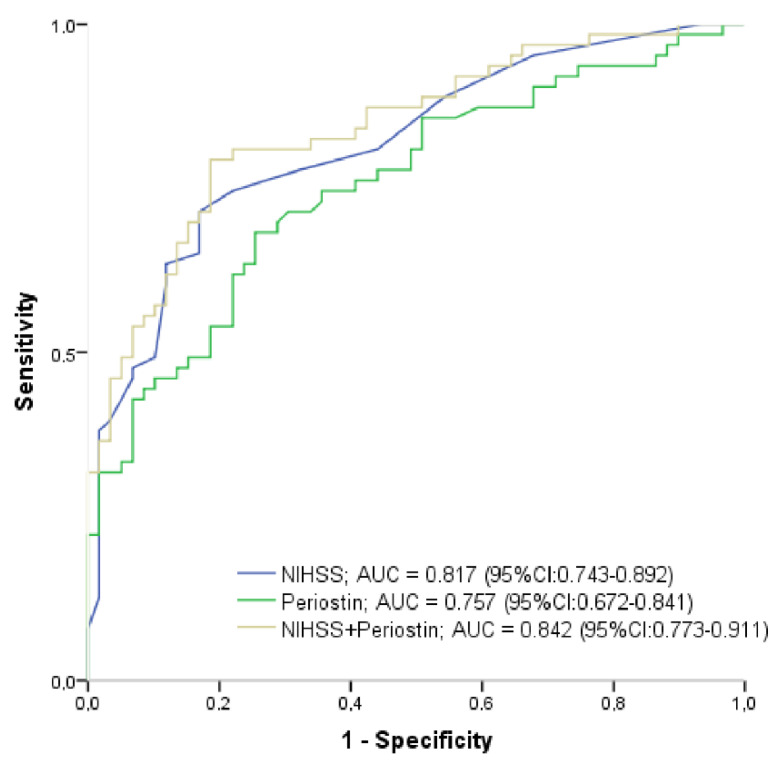
Receiver operating characteristic curve analysis of prognostic predictive ability of serum NIHSS score on admission, serum level of periostin on admission and the combination of NIHSS score and periostin for 3-month unfavorable outcome in patients with acute ischemic stroke. AUC, area under the curve; CI, confidence interval; NIHSS, National Institute of Health Stroke Scale.

ASPECT score < 6 indicating patients with poor collaterals on admission was positively associated with admission GCS in univariate analysis. In contrast, diabetes, atrial fibrillation, admission NIHSS, periostin level, NLR, CRP as well as creatinine levels were inversely associated with ASPECT score < 6 calculated in univariate analysis. Age and sex were not shown correlation with ASPECT < 6. Although GCS, NIHSS, atrial fibrillation, CRP, diabetes, creatinine and NLR were adjusted in a binary logistic regression model, serum periostin level remained a significant predictor for ASPECT < 6 status on admission (OR, 5.911; CI, 0.990–0.999; *p* = 0.015, Table 3). Next, another binary logistic regression analysis was performed to explore independent predictors of the outcome: NIHSS on admission and atrial fibrillation were independently associated with a favorable 90-day outcome (mRS0–2). Based on ROC analysis, both NIHSS score (AUC, 0.817; 95% CI, 0.743–0.892, cutoff: 8.5, sensitivity: 75%, specificity: 78%, *p* < 0.001) and admission serum concentration of periostin (AUC, 0.757; 95%CI, 0.672–0.841, cutoff: 466.7 ng/L, sensitivity: 75 %, specificity: 65%, *p* < 0.001) showed similar sensitivity and specificity in the prediction of unfavorable 3-month outcome. In contrast, the combination of these two variables had significantly greater predictive power (AUC, 0.842; 95% CI, 0.773–0.911, *p* < 0.001) (Figure 3). The serum concentration of periostin with a cut-off value of ≥594.5 independently predicted admission ASPECT < 6 reflecting the poor collateral status with a sensitivity of 84.2% and specificity of 72%.

## 4. Discussion

As a novelty, we investigated the matricellular protein periostin in humans in the hyperacute phase of acute ischemic stroke. The major findings were the following: (i) periostin level measured within 6 h after onset of the index event was significantly elevated in patients with unfavorable outcome at 90-day follow-up; (ii) ASPECT score reflecting the collateral circulation was negatively, while NIHSS indicated the severity of IS was positively correlated with systemic concentration of periostin; (iii) finally, periostin level measured on admission was independently associated with ASPECT score < 6 calculated on admission CT scan. Thus, serum periostin level at the time of admission indirectly indicates the quality of the collateral network in the acute phase of ischemic stroke.

Up to now, a growing evidence has suggested that periostin was expressed in high levels in primates and can enhance neurite outgrowth activity of the surrounding neurons [18]. Moreover, periostin significantly enhances neural stem cell proliferation and differentiation after hypoxic-ischemic injury [19]. The upregulation of periostin was also reported in a cerebrovascular clinical setting and in neuronal injury by head trauma. Dong et al. found that serum periostin concentration on admission was an independent predictor for 30-day mortality and overall survival after head trauma [20]. Another study by Ji et al. provided evidence that periostin concentrations of the sera from intracranial hemorrhage (ICH) patients were highly correlated with hematoma volume and baseline NIHSS scores; and serum periostin emerged as an independent predictor for 6-month unfavorable outcome after acute ICH [21]. Moreover, serum periostin concentrations resembled hematoma volume and NIHSS score in terms of predictive value assessed by AUCs for 6-month unfavorable outcome [21]. Lu et al. obtained similar results in a study of subarachnoid hemorrhage, with serum periostin levels showing an independent association with poor outcome and DCI [10]. Serum periostin levels were increased at 6 days and beyond at 4 weeks post-ischemia and were positively correlated with severity in terms of infarct volume and neurological deficit, but not with functional outcome in patients with large-artery atherosclerotic stroke [11]. Interestingly, in this study the periostin level measured on the first day showed no difference compared to the values measured in the control group. In contrast, in our study, serum periostin levels measured in the hyperacute phase of IS were increased compared to healthy controls. Importantly, our study population shows several differences: (i) in ethiology, as almost one-third of the total study population and 43% of the unfavorable outcome subgroup presented with atrial fibrillation on admission; (ii) inflammatory status, as CRP an NLR were higher in patients with poor 90-day outcome; and (iv) metabolic status, as plasma glucose level was significantly elevated among patients with poor outcome. In accordance with He et al., serum concentration of periostin showed strong correlation with NIHSS in our cohort. Additionally, we also find the relationship between systemic concentration of periostin measured in the acute phase of stroke and ASPECT score a valid assessment of the collateral circulation of the ischemic brain. Taken together, these may suggest that matricellular proteins (e.g., apelin, oncostatin M, and periostin) regulating myogenesis/angiogenesis and vessel formation during regeneration processes [22] may have an impact on the collateral circulation determining the salvageable penumbral brain tissue [23].

Based on the aforementioned clinical studies, periostin emerged as a novel prognostic marker in assessing the long-term outcome ofdiseases with severe neurological damage. However, there is a great need to find markers estimating the cerebral collaterals upon developing an acute ischemic insult. Periostin as a prehospital point-of-care marker might have the potential to provide information about the prior collateral status and theexpected outcome, contributing to early diagnostics and the clinical decisionmaking of the stroke care team. In our study, we found that serum periostin concentration is an independent predictor of ASPECT < 6 calculated on admission, and this finding indicates that periostin could reflect the extent of early brain injury in hyperacute stage of ischemic stroke. According to the European Stroke Organisation—European Society for Minimally Invasive Neurological Therapy (ESMINT) Guidelines on Mechanical Thrombectomy in Acute Ischemic Stroke [24], patients with AIS with large vessel occlusion should be treated with mechanical thrombectomy plus best medical management up to approximately 7 h 18 min after stroke onset, without the need for perfusion imaging-based selection if patients have ASPECTS ≥ 6 and moderate-to-good collateral circulation. Since many studies [12,13,14,15,16] prove that ASPECT score is a sensitive marker of the ischemic core, and thus indirectly of the collateral network, its close correlation with periostin can act as an important diagnostic tool. Considering this, periostin as a surrogate marker measured within 6 h after acute stroke might contribute to establish the indication of mechanical thrombectomy in the lack of advanced neuroimaging.

However, the source of elevated periostin is not yet clear. Periostin is generally present at low levels in most adult tissues, but is highly expressed at sites of injury or inflammation and in tumors of adult organisms. Current evidence demonstrates that periostin actively contributes to tissue injury, inflammation, fibrosis and tumor progression [4]. Therefore, these extracerebral sources should be excluded prior to any therapeutic decisions. Given the acute increase in periostin level in our study in patients with poor outcome and ASPECT < 6, it is possible that the acute breakdown of the blood–brain barrier and leakage into circulation may be a significant source. Liu et al. demonstrated that periostin was upregulated in cerebral cortex after experimental SAH in mice and was responsible for early brain injury, which was possibly mediated by p38/ERK/MMP-9 signaling pathways [25]. Furthermore, in our cohort, CRP, NLR, and WBC showed a close correlation with admission periostin levels, indicating a possible link between the early immune response and post-ischemic brain injury. C-reactive protein and NLR have recently been reported as potential novel biomarkers of the baseline inflammatory process and could serve as outstanding predictors in patients with ischemic stroke [26]. Elevated levels of CRP after stroke have been related to poor functional outcome and mortality [27]. In asthma, periostin is recognized as a biomarker of type 2 inflammation and periostin (POSTN) gene expression is up-regulated in bronchial epithelial cells by IL-13 and IL-4 [6]. Our study also found a strong positive association between atrial fibrillation and the admission level of periostin. As a matricellular protein, periostin has been proved to play an important role in fibrogenesis [28,29]. Wu et al. found that the upregulated expression of transforming growth factorβ (TGF-β) and periostin in renin–angiotensin–aldosterone system (RAAS) is correlated with the degree of atrial fibrosis in patients with AF [30]. Furthermore, evidence of a recent review supports the potential role of periostin in the pathophysiology of cardiovascular diseases [31]. These findings suggest that the source of perositin, in addition to brain tissue damage that enters the systemic circulation through damage to the blood–brain barrier, may also be of myocardial origin.

Summarizing, initial periostin level may serve as a surrogate prognostic marker for hyperacute ischemic stroke reflecting stroke severity, long-term outcome, and patient’s eligibility for intervention.

However, our study has several limitations. First, data about the changes of circulating periostin concentrations during the progression of ischemic stroke were not reported. Periostin kinetics could provide important information regarding the evolution of stroke. Second, only native and CT angiography were performed on admission, while MR would have been more suitable to determine the size of early infarction. As CT angiography-based collateral score calculation was not performed in all cases, we were not able to include it into our study. Third, various isoforms of periostinhave been described in humans [9]; the ELISA kit used in our study detects all periostin isoforms present in the circulation and was not capable to differentiate between them. Fourth, we did not measure other conventional markers of both brain and heart tissue damage such as S100B or troponin, so it is unclear whether the measured periostin level is entirely of cerebral origin [32]. In addition, the relatively small number of cases in our study limits the generalizability of the study results. Therefore, further studies are warranted to explore the real specificity of periostin in early brain damage due to ischemia. As the pathophysiology, prognosis and clinical features of acute small vessel ischemic strokes are different from other types of cerebral infarcts, anethiology-based intergroup comparison of systemic concentration of periostin would be worthwhile in the future [33].

In conclusion, there is an urgent need to find reliable markers reflecting the collateral circulation in patients with acute IS either predicting the extension of early brain damage or supporting decision-making in cases where opportunities for advanced neuroimaging are limited. Reasonably, the concept of endothelial dysfunction as well as other vascular features related to cerebrovascular insults might have to be redefined as we learn more about multiple proteins (e.g., matricellular proteins) secreted by endothelial cells besides nitrogenoxide [34,35]. Overall, periostin may be a useful laboratory marker to identify patients with a poor collateral network and consequently with a more extensive core, especially when advanced neuroradiological procedures, e.g., CT perfusion, are not available. If randomized controlled studies with a higher number of cases confirm our results, measuring periostin serum levels could be developed into a point-of-care diagnostic test. Thus, the prehospital selection of patients suitable for neurointervention could be significantly improved.

## Figures and Tables

**Table 1:** Baseline characteristics of the study population. Continous variables are expressed as medians (interquartile ranges). Categorical values are given as frequencies (percentages). Abbreviations: GCS, Glasgow coma scale; NIHSS, National Institutes of Health Stroke Scale; SBP, systolic blood pressure; DBP, diastolic blood pressure; ASPECTs, Alberta stroke programme early CT score; WBC, white blood cell; NLR, neutrophil–lymphocyte ratio; CRP, C-reactive protein; tPA, tissue plasminogen activator; * at 90-day follow-up.

Characteristics	Total (n = 122)	Favorable * Outcome (n = 59)	Unfavorable * Outcome (n = 63)	*p*-Value
Age, y, median (IQR)	71 (63–79)	71 (62–77)	73 (64–79)	0.127
Male, n (%)	74 (60.7%)	35 (59.3%)	39 (61.9%)	0.770
Hypertension, n (%)	100 (82%)	48 (81.4%)	52 (82.5%)	0.865
Diabetes, n (%)	35 (28.7%)	17 (28.8%)	18 (28.6%)	0.976
Smoking, n (%)	52 (38%)	19 (32.2%)	33 (52.4%)	0.024 *
Atrial fibrillation, n (%)	34 (27.9%)	7 (11.9%)	27 (42.9%)	<0.001 *
Large artery atherosclerosis, n (%)	59 (48.4%)	35 (59.3%)	24 (38.1%)	0.029
Lacunar, n (%)	23 (18.9%)	13 (22%)	10 (15.8%)	0.385
Other, n (%)	4 (3.3%)	3 (5.1%)	1 (1.6%)	0.278
Undetermined, n (%)	2 (1.5%)	1 (1.7%)	1 (1.7%)	0.899
GCS, median (IQR)	15 (12–15)	15 (15)	14 (11–15)	<0.001 *
NIHSS, median (IQR)	8 (5–16)	6 (4–8)	13 (8–19)	<0.001 *
SBP, median (IQR)	150 (130–170)	148 (130–170)	160 (138–180)	0.237
DBP, median (IQR)	84 (80–94)	82 (80–90)	86 (80–100)	0.463
ASPECTs, median (IQR)	9 (7–10)	10 (9–10)	8 (6–9)	<0.001 *
WBC, median (IQR)	8.4 (6.9–10.7)	7.7 (9–10)	8.8 (7–11)	0.264
NLR, median (IQR)	2.9 (2–5.6)	2.5 (1.7)	3.6 (2.5–7.3)	0.002 *
platelet, median (IQR)	242 (188–306)	245 (196–300)	238 (185–305)	0.625
creatinine, median (IQR)	86 (73–102)	83 (70–97)	87 (74–104)	0.411
glucose, median (IQR)	7.2 (6.2–8.9)	6.8 (5.9–8.1)	7.8 (6.8–9)	0.004 *
CRP, median (IQR)	3.7 (1.4–9.5)	2.6 (1.4–5.4)	5.1 (1.7–16)	0.042 *
Thrombectomy, n (%)	29 (23.8)	14 (23.7)	15 (23.8)	0.856
Intravenous tPA, n (%)	51 (41.8)	28 (47.5)	23 (36.5)	0.190
Thrombectomy plus intravenous tPA, n (%)	17 (13.9)	6 (10.2)	11 (17.5)	0.260
Conservative, n (%)	25 (20.5)	11 (18.6)	14 (22.2)	0.658
serum level of periostin, median (IQR), ng/L	462 (297–735)	390 (260–563)	615 (443–1070)	<0.001 *

**Table 2:** Spearman correlation between admission clinical parameters and serum periostin level measured at 24 h after admission. Coefficient (r) values > 0 indicate a positive association; values < 0 indicate a negative association. Statistically significant values are given in bold. ASPECT, Alberta stroke program early CT score.

Variable	Spearman Correlation Coefficient (r)	*p*-Value
Atrial fibrillation	0.335	**<0.001**
Systolic blood pressure	0.068	0.459
Diastolic blood pressure	0.119	0.193
Glasgow Coma Scale	−0.308	**<0.001**
ASPECT score	−0.590	**<0.001**
White blood cell count, G/L	0.239	**0.01**
Neutrophil-lymphocyte ratio	0.328	**<0.001**
Creatinine, µmol/L	0.277	**0.003**
C-reactive protein, mg/L	0.285	**0.002**
Glucose, mmol/L	0.257	**0.007**
Platelet, G/L	−0.059	0.534
Carbamide, mmol/L	0.245	**0.01**

**Table 3:** Binary logistic regression analysis of predictors for admission ASPECT score < 6 in patients with acute ischemic stroke.CI, confidence interval. Model 1 included Glasgow Coma Scale and National Institute of Health Stroke Scale. Model 2 included variables in model 1 plus atrial fibrillation and admission level of C-reactive protein. Model 3 included variables in model 2 plus diabetes, admission serum level of creatinine and admission neutrophil-lymphocyte ratio.

	Odds Ratio	95% CI	*p*-Value
periostin	15.532	0.995–0.998	<0.001
Model 1	6.339	0.995–0.999	0.012
Model 2	5.917	0.993–0.999	0.015
Model 3	5.911	0.990–0.999	0.015

## Data Availability

The data presented in this study are available on request from the corresponding author.
